# Living Well with Diabetes: a randomized controlled trial of a telephone-delivered intervention for maintenance of weight loss, physical activity and glycaemic control in adults with type 2 diabetes

**DOI:** 10.1186/1471-2458-10-452

**Published:** 2010-08-03

**Authors:** Elizabeth G Eakin, Marina M Reeves, Alison L Marshall, David W Dunstan, Nicholas Graves, Genevieve N Healy, Jonathan Bleier, Adrian G Barnett, Trisha O'Moore-Sullivan, Anthony Russell, Ken Wilkie

**Affiliations:** 1 The University of Queensland, Level 3 Public Health Building, School of Population Health, Cancer Prevention Research Centre, Herston Road, Herston, QLD, Australia; 2 Queensland University of Technology, School of Public Health, Faculty of Health, Brisbane, Australia; 3 Baker IDI Heart and Diabetes Institute, Melbourne, Australia; 4 The University of Queensland, Centre for Diabetes and Endocrine Research, Princess Alexandra Hospital, Brisbane, Australia; 5 Southeast Primary Healthcare Network, Logan, Australia

## Abstract

**Background:**

By 2025, it is estimated that approximately 1.8 million Australian adults (approximately 8.4% of the adult population) will have diabetes, with the majority having type 2 diabetes. Weight management via improved physical activity and diet is the cornerstone of type 2 diabetes management. However, the majority of weight loss trials in diabetes have evaluated short-term, intensive clinic-based interventions that, while producing short-term outcomes, have failed to address issues of maintenance and broad population reach. Telephone-delivered interventions have the potential to address these gaps.

**Methods/Design:**

Using a two-arm randomised controlled design, this study will evaluate an 18-month, telephone-delivered, behavioural weight loss intervention focussing on physical activity, diet and behavioural therapy, versus usual care, with follow-up at 24 months. Three-hundred adult participants, aged 20-75 years, with type 2 diabetes, will be recruited from 10 general practices via electronic medical records search. The Social-Cognitive Theory driven intervention involves a six-month intensive phase (4 weekly calls and 11 fortnightly calls) and a 12-month maintenance phase (one call per month). Primary outcomes, assessed at 6, 18 and 24 months, are: weight loss, physical activity, and glycaemic control (HbA1c), with weight loss and physical activity also measured at 12 months. Incremental cost-effectiveness will also be examined. Study recruitment began in February 2009, with final data collection expected by February 2013.

**Discussion:**

This is the first study to evaluate the telephone as the primary method of delivering a behavioural weight loss intervention in type 2 diabetes. The evaluation of maintenance outcomes (6 months following the end of intervention), the use of accelerometers to objectively measure physical activity, and the inclusion of a cost-effectiveness analysis will advance the science of broad reach approaches to weight control and health behaviour change, and will build the evidence base needed to advocate for the translation of this work into population health practice.

**Trial Registration:**

ACTRN12608000203358

## Background

### Type 2 diabetes - a population health problem

The rapid increase in the prevalence of type 2 diabetes, obesity and associated complications is a major public health problem. In Australia, data from the 1999-2000 AusDiab study estimated that approximately 1 million (7.4%) Australian adults aged 25 years and over have type 2 diabetes [1], with approximately 85% being overweight or obese [2]. The growing epidemics of type 2 diabetes and obesity have been concurrent, with the prevalence of both doubling over the past 20 years [1]. The prevalence of type 2 diabetes is predicted to increase dramatically over the next few decades; projections are that by 2030, 8.4% Australian adults aged 20-79 will have the disease [3]. Similar rapidly increasing prevalence estimates have been reported for the UK [3], USA [3,4] and other developed and developing countries [3].

Identified as the seventh leading cause of death among Australian adults, type 2 diabetes is a major cause of premature mortality and morbidity due to cardiovascular, renal, ophthalmic and neurological disease [5]. Data from the DiabCost Australia study, which assessed the total costs of type 2 diabetes to the health care system, society, government and carers, estimated that $3 billion a year is spent treating type 2 diabetes [6]. Since the burden of illness associated with type 2 diabetes increases with the onset of microvascular and macrovascular complications [6], there is high priority for effective and sustainable strategies to slow or prevent the onset of complications. The value of achieving glycaemic control is underscored by the landmark findings of the UK Prospective Diabetes Study, a 10 year observational follow-up in patients with diabetes, whereby each 1% reduction in glycosylated haemoglobin (HbA1c) was associated with a 14% reduction in myocardial infarction, a 37% reduction in microvascular events, and a 21% reduction in diabetes-related mortality [7]. This is of particular importance for people with type 2 diabetes since coronary heart disease is responsible for about 70% to 80% of deaths in this population [8].

Lifestyle factors that adversely affect energy balance (physical inactivity and over-nutrition) play a major role in the development of both type 2 diabetes and obesity [9,10]. The importance of weight management, by means of a healthy, energy-restricted diet and a physically active lifestyle, is considered to be the primary approach in the treatment of overweight and obesity [11,12] and is widely acknowledged as a cornerstone of the management of type 2 diabetes and its related morbidities [13]. There is also evidence that many of the traditional pharmacologic approaches frequently used to treat diabetes contribute to weight gain [14]. Weight loss or weight management is an important therapeutic task for most people with type 2 diabetes, not only for improved glycaemic control but also for reducing cardiovascular disease (CVD) risk [13]. A modest weight loss of 5-10% of body weight has been associated with improvements in fasting plasma glucose, HbA1c, insulin, lipid levels, and blood pressure [10]. In addition, there is compelling epidemiologic and experimental evidence that physical activity confers substantial protection against CVD and premature mortality in those with type 2 diabetes, independent of weight loss, through its favourable effects on blood pressure, blood glucose, insulin sensitivity, lipid profile, fibrinolysis, endothelial function and inflammatory defence systems [15,16].

At present in Australia, type 2 diabetes is managed primarily in the general practice setting (primary care). In addition to medication management and monitoring of glycaemic control, this may include brief lifestyle advice from General Practitioners (GPs), and for some, a Diabetes Care Plan that includes referral for a limited number of visits to diabetes educators, dietitians, exercise physiologists and other allied health professionals. Given the challenge of health behaviour change and weight loss, and the need for ongoing assistance to maintain such changes, approaches that work in concert with primary care are needed, with such approaches involving more behaviourally-focussed and longer-term self-management supports [17].

### Lifestyle management and weight loss intervention trials in type 2 diabetes

There is a large literature on lifestyle management and weight loss interventions in type 2 diabetes, including the current landmark Look AHEAD trial, which is evaluating an intensive four-year weight loss intervention to reduce cardiovascular morbidity and mortality [18,19]. Across numerous studies, there is clear evidence that intensive lifestyle interventions involving reduced energy (and fat) intake, regular physical activity, cognitive behaviour therapy and frequent participant contact will produce significant behavioural improvements, as well as weight loss (5-7% of body weight) and concomitant improvements in glycaemic control and dyslipidaemia [10,20,21]. However, there are a number of gaps in this literature that make its findings less relevant to informing a population-based approach to the lifestyle management of type 2 diabetes. First, the majority of trials have evaluated intensive, clinic-based interventions that are similar to the diabetes education programs offered through hospitals as part of clinical management. While these clinic-based interventions are effective [22], few patients with type 2 diabetes take part in such programs [23]. Second, very few studies include a post-intervention follow-up assessment to determine whether improvements are maintained longer term [24,25].

### Broad reach weight loss and lifestyle intervention trials

A number of recent studies have evaluated weight loss interventions delivered via telephone, tailored print and the internet [26-31]. Of note are two recent studies which have evaluated a telephone-delivered approach for initiation and maintenance of weight loss [29,31]. One study observed similar short-term weight loss at 6-months for both face-to-face (8.9%) and telephone-delivered (7.7%) interventions [29]. In the other trial, following initial weight loss at 6 months, small but statistically non-significant regain in weight was observed for those in the 12-month telephone and face-to-face maintenance contact groups, which was significantly less than the regain observed in the control group [31].

Telephone-delivered interventions have the potential for widespread and cost-effective population reach, and have been widely researched [32-38]. A systematic review of 26 telephone-delivered physical activity and dietary behaviour intervention studies found very strong support for their efficacy to produce short-term behavioural changes in both people with and without chronic conditions, with 20 of 26 studies reporting significant behavioural improvements immediately post-intervention [39]. Interventions lasting six to 12 months and those including 12 or more calls produced the most favourable outcomes. Our own work in this area has shown telephone-delivered interventions to be effective in promoting both initiation and maintenance [40,41] of dietary change, as well as demonstrating cost-effectiveness [42].

### Purpose

This paper describes the methods of the Living Well with Diabetes trial which is evaluating a telephone-delivered behavioural weight loss intervention focussing on physical activity, diet and behavioural therapy in adults with type 2 diabetes. To promote maintenance the 18 month intervention has an intensive first 6 months focussed on *initiation *of behaviour change, followed by 12 months focussed on enhancing/supporting *maintenance*. Measurement at baseline, six, 12, 18, and 24 months allows for assessment of *initiation *and *maintenance *of change - a major contribution of the study to the evidence base on broad reach interventions to support weight loss and lifestyle management in type 2 diabetes.

## Methods/Design

### Study design

Living Well with Diabetes is a two-arm randomised controlled trial evaluating a telephone-delivered behavioural weight loss intervention versus usual care in adults with type 2 diabetes. Ethical approval was granted from The University of Queensland Behavioural and Social Sciences Ethical Review Committee.

### Setting

Participants are recruited from general practices in the city of Logan (population 270,000), a large ethnically and socioeconomically diverse community in Queensland, Australia, which sits 35 kilometres from Brisbane (the state capital), and an urban centre of 1.8 million residents.

### Practice sampling and recruitment

General practices are recruited from the Southeast Primary Health Care Network (SPHN), a state and federally funded organization that provides administrative, technical and professional development/educational support to local area practices. At study commencement, SPHN supported 76 practices. The study will recruit 10 practices over two years, with approximately 30 adults enrolled from each practice. To ensure an adequate patient sampling frame from each practice, practices are ranked in order of descending number of GPs, with the largest practices approached first on a rolling basis until 10 practices (or the required sample size of 300 participants) consent to participate. Practices are initially contacted by telephone to determine whether electronic medical records can be used to perform patient searches by medical condition, and whether approximately 200 patients with type 2 diabetes can be identified. Eligible practices are sent an invitation letter and study brochure. This is followed-up by a phone call from a GP working within the SPHN (author KW), and a practice visit from the project manager to solicit consent.

### Patient sampling and recruitment

Within practices, electronic medical records are searched for potentially eligible patients. Eligibility is based on: diagnosed type 2 diabetes; aged 20-75 years; and having a listed telephone number. The lists of potentially eligible participants are initially screened by treating GPs for the following contraindications to an unsupervised physical activity and weight loss intervention: active heart disease, breathing problems requiring hospitalisation in the past 6 months, undergoing dialysis, taking Warfarin, diabetic complications such as severe neuropathy or retinopathy, planning a knee or hip replacement in the next 12 months, regular use of a mobility aid or pregnant. In addition, GPs are asked to screen out those who are undergoing cancer treatment (excluding hormone therapy), those from non-English speaking backgrounds and those with significant difficulty with written materials or with hearing problems that would make telephone contact prohibitive.

Eligible patients, based on GP screening, receive a letter from their GP informing them of the study, along with a study brochure and an expression of interest form with a return, pre-paid envelope. Patients not returning the form within two weeks receive a follow-up phone call from practice staff. Those patients returning the expression of interest form and those providing verbal consent to contact are posted a study information sheet and consent form. They then receive a call from study staff during which a detailed explanation of the study is provided, patients are re-screened for eligibility based on the same criteria as GPs (to account for variation in GP screening), and some additional exclusion criteria - current or planned (next 12-months) use of weight loss medications (e.g. Xenical, Reductil), and previously had or planning bariatric surgery. During this call, participants are also screened for self-reported baseline levels of physical activity and weight, and are excluded from study participation if they meet or exceed physical activity guidelines (30 minutes of moderate to vigorous physical activity on five or more days/week [43] AND are not overweight (defined as a body mass index [weight in kilograms/height in metres^2^] of < 25.0 kg/m^2^) [44]. In addition, for those with a self-reported BMI ≥ 45 kg/m^2^, suitability for participation in unsupervised weight loss is confirmed from their GP. Consent to participate in the study is then sought from those considered eligible. De-identified data on eligible non-participants is obtained from medical records to compare demographic (age and sex) characteristics of participants and non-participants.

### Randomisation

Following baseline data collection, allocation to study groups (telephone intervention or usual care) is conducted using the method of minimization to ensure equal group balance across factors likely to impact on primary study outcomes [45,46]. Minimisation factors based on baseline data collection include: gender, age (< 55 years; ≥ 55 years), BMI (< 40 kg/m^2^; ≥ 40 kg/m^2^), HbA1c (< 8%; ≥ 8%), physical activity level (meeting guidelines of at least 150 minutes/week and 5 or more sessions; not meeting guidelines) [47], and diabetes management (insulin or combination therapy; traditional oral hypoglycaemic medications; new glucose-lowering medications (e.g. Byetta, Januvia); lifestyle alone). Allocation is performed using the free Minim computer software [48] and conducted by a research assistant with minor involvement in participant recruitment. The timeframe from participant recruitment to group allocation ranges from approximately three to eight weeks to allow for extensive baseline data collection (see Data Collection section below), and not all minimisation factors are known at recruitment (e.g. HbA1c level, diabetes management). Therefore, the ability for the staff member to predict with certainty, the allocation of participants at recruitment, a criticism of the minimisation method [46], is near impossible.

### Usual care

In order to minimise attrition over the 24-month study duration, participants in the usual care group receive a thank you letter and brief summary following each assessment. At baseline, 6-, 12- and 18-months they are also posted standard, off-the-shelf diabetes self-management education brochures (from Diabetes Australia) with information on diabetes education and a variety of health behaviours. These are consistent with the materials generally given to patients as part of their usual diabetes care. GPs for all patients in the study are also sent brief summaries of their patients' assessment results.

### Telephone-delivered weight loss intervention

The weight loss intervention, delivered entirely over the telephone, uses a combined approach of increasing physical activity, reducing energy intake and behavioural therapy [11,12]. It is based on current evidence for initiation and maintenance of weight loss and clinical practice guidelines for the management of overweight/obesity and type 2 diabetes. Participants receive a detailed workbook at the commencement of the intervention and up to 27 telephone calls over 18 months, to support both initiation and maintenance of weight loss. Call frequency is somewhat flexible, so that it can be tailored to the needs of the participant. For example, an extra weekly call might occur during a period of relapse.

Intervention targets for physical activity, dietary intake and weight loss are consistent with management goals for type 2 diabetes [10], with the aim to reduce glycosylated haemoglobin (HbA1c) to less than 7% [11,49,50]. As described in detail below, participants work with their telephone counsellor to identify small, gradual changes to physical activity and dietary intake and that are able to be maintained long-term, rather than large and highly restrictive changes which are less likely to be maintained [11,51], as described below. Participants are encouraged to aim for moderate weight loss of 5-10% of initial body weight [10,21], with a loss of 1-2 kg per month.

#### Physical activity

A target of 210 minutes per week (30 minutes every day) of moderate-intensity, planned activity is encouraged, consistent with the level of physical activity necessary to promote weight loss [49]. Given evidence suggesting that even higher levels of activity (i.e., 60 minutes/day) may be necessary for weight loss maintenance [52], those who can do more activity will be encouraged to work towards this during the maintenance phase. The telephone counsellor works with participants to first identify types of activities that they enjoy and can be easily incorporated into their lifestyle (e.g., walking), rather than providing a structured exercise program [11,53]. As most participants are relatively inactive at baseline, increases in physical activity are initially small and gradually increase towards the goal of 30 minutes per day of moderate-to-vigorous intensity activity [49].

Resistance exercise (2-3 sessions/week) is also encouraged, as strength training has benefits for glycaemic control [54] and is consistent with the physical activity guidelines for older adults [55]. Participants are provided with a resistance band, and detailed photographs and instructions are included in the workbook with guidelines on the number of sets and repetitions of each exercise, along with options for progression.

In addition to daily planned physical activity, participants are encouraged to take opportunities to be active in and around their homes and workplaces (e.g. gardening, housework, taking the stairs), and to reduce sitting time (i.e., to get up and move every 30 minutes and to aim for no more than 2 hours/day of screen time outside of work). This is consistent with evidence on the health benefits conferred by light-intensity activity [56-58], and with newer evidence on the cardio-metabolic consequences of too much sitting [59,60].

#### Diet

Participants are encouraged to reduce energy intake by 2,000 kJ per day through individualised advice to allow for specific food preferences and approaches [10,12,61]. That is, the intervention does not use meal replacements or structured meal plans. Specific macronutrient compositions are not prescribed (e.g. high protein/low carbohydrate or high carbohydrate/moderate protein) as evidence on long-term maintenance of weight loss has shown similar outcomes for both types of diet [62-64]. A low fat diet (< 30%) is encouraged as this has been associated with long-term weight loss maintenance [52], as are a saturated fat intake of < 7% of energy and fibre intake of 25 grams/day for women and 30 grams/day for men, consistent with dietary recommendations for people with type 2 diabetes to reduce risk factors for cardiovascular disease [65]. Strategies to reduce energy intake include - improving portion control (by reducing portion size or number of serves) and lowering energy density (by increasing intakes of low energy foods such as fruits and vegetables and reducing intake of energy dense foods such as high fat foods). Regular meal patterns with an emphasis on eating breakfast are promoted [66].

#### Behavioural therapy

Behaviour change strategies and principles used to guide the intervention are derived from Social Cognitive Theory (SCT) [18], specifically developing knowledge and skills related to the SCT constructs of self-efficacy, social support and outcome expectancies. The matrix in Table 1 and the text below describe the way in which these are operationalized. No single behaviour strategy has been shown to be better than any other in terms of effects on weight loss, and thus multi-modal strategies are used [11,12]; although some strategies (i.e., self-monitoring) have been associated with better weight loss maintenance outcomes [67]. Behaviour change skills emphasised in this intervention include: self-monitoring, goal setting, problem solving, social support, stimulus control, positive self-talk, and self-reward.

**Table 1:** Theory matrix

Social-Cognitive Theory Constructs	Operationalization
Self-efficacy	Realistic and measurable goal-setting
	Assessing confidence
	Self-monitoring
	Practicing positive self-talk
	
Social support	Developing a support network (family/friends/others)
	Setting goals around using supports
	
Outcome expectancies	Benefits and barriers to health behaviour change
	Problem-solving approach to addressing barriers
	Rewards for goal attainment

Participants are provided with self-monitoring 'trackers,' to monitor their daily physical activity and food intake, and are encouraged to weigh themselves once to twice weekly. Participants are provided with a pedometer to monitor daily steps and with a set of digital scales to monitor their body weight.

Participants are encouraged to set small, measurable and achievable health behaviour change and weight loss goals that facilitate a sense of confidence and mastery that can be built upon throughout the intervention and hence build self-efficacy. These are recorded on a large, reusable refrigerator magnet. Problem solving is used to help participants identify barriers to their behaviour change goals and identify strategies and solutions to overcome these.

Participants are encouraged to identify a broad array of possible supports for weight loss (e.g., from family and friends co-workers, the general practitioner, neighbourhood or community programs) and to set specific goals for drawing upon these [68]. Stimulus control (or behaviour cue) techniques are used to create more supportive environments for health behaviour change and weight loss (e.g. keeping problem foods out of the house; putting left-overs in the freezer immediately; leaving walking shoes by the bedside). Positive self-talk (e.g. focussing on what has been achieved rather than what has been given up) is encouraged as a means of addressing habitual negative thinking [69], and self-rewards for goal attainment are promoted to reinforce behaviour change.

#### Telephone counsellor background and training

All telephone counsellors have at least bachelor's level training in nutrition and dietetics; some have also completed a dual-degree in exercise physiology. They undergo one month of intensive training in study protocols and a motivational interviewing approach to health behaviour counselling [70] (including role plays with study investigators). Telephone counsellors have ongoing quality assurance checks via randomly taped telephone calls and fortnightly clinical supervision meetings. In addition, call attempts, completions and duration are tracked, and a call content checklist completed after each call to inform an evaluation of intervention fidelity. To the extent possible, the same telephone counsellor remains with participants throughout the duration of the intervention to facilitate rapport and continuity of care.

#### Intervention procedures

**Figure 1:**
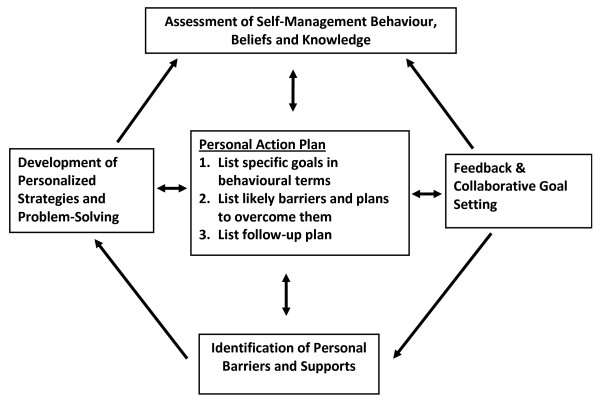
**Chronic Disease Self-Management Intervention Model**.

Intervention delivery is guided by a patient-centred, chronic disease self-management intervention model used in previous trials (Figure 1) [37,71], and by techniques of motivational interviewing [70]. An iterative process is used in each call: it draws upon repeated *assessment *of study outcomes and participant self-monitoring; *feedback *is provided in relation to study targets for weight, dietary intake and physical activity, and consistent with a motivational interviewing approach, this feedback highlights the discrepancy between participant goals and their current health behaviours; *collaborative goals *for weight, physical activity and dietary change are set with the telephone counsellor, with an emphasis on achievability and measurability. Each intervention contact results in a *behaviourally-specific Action Plan *that specifies exactly what is to be done and when; *barriers and supports *are identified; *confidence *is assessed and *problem-solving *is discussed as necessary. These steps are repeated during intervention calls, with goals being adjusted as necessary.

A semi-structured approach to both the content and the order in which intervention targets are addressed is used to guide intervention delivery (see Table 2). However, consistent with the motivational interviewing approach, the intervention is tailored to each participant, with an initial focus on targets in which she/he is most motivated and most confident in changing.

**Table 2:** Telephone Intervention Overview: Call Frequency and Content

Intervention stage & call frequency	Purpose	Content
**Phase 1**: Month 1 4 weekly calls	Building rapport	Program introduction/explanation Participant overarching program goals Feedback on baseline assessment Review & goal-setting: *Weight loss*: benefits, energy balance, realistic targets, portion sizes, recommended serves, reducing fat intake Behaviour change skills: self-monitoring, goal-setting Begin use of scale, pedometer and food diary

**Phase 2**: (Months 2-6) 11 fortnightly calls	Behaviour change Weight loss Skill-building	Review & goal-setting: *Physical activity*: planned activity, incidental activity, sitting time, strength training *Diet*: reducing energy intake (choosing lower energy foods, swapping higher energy foods for lower energy foods, decreasing intake of high energy foods/drinks), eating habits, food labels, modifying recipes Behaviour change skills: rewards, behavioural cues, barriers and supports Progress review at 3 months

**Phase 3**: (Months 7-18) 12 monthly calls	Maintenance of behaviour change & weight loss	Feedback on 6-, 12- & 18-month assessments Progress review at 9 & 15 months Review & goal-setting: Role of physical activity in maintaining weight loss Problem-solving Social-environmental support Developing lifelong habits

##### Phase 1 (month 1)

The first month of the intervention involves up to four weekly calls, with a focus on building rapport (e.g. providing feedback on the baseline assessment and understanding what the participant wants to get out of the program); education on weight loss (e.g. energy balance); and skill-building (e.g. self-monitoring and goal-setting). The emphasis is on helping participants to gain the knowledge and skills necessary to achieve behaviour change and weight loss in the next phase.

##### Phase 2 (months 2-6)

Up to 11 fortnightly calls are completed during this phase, with an emphasis on initial behaviour change, weight loss and continued confidence and skill-building. Behaviourally-specific goal setting with regard to the amount of physical activity and dietary change that is needed to achieve weight loss is the focus, along with problem-solving and continued self-monitoring.

##### Phase 3: (months 7-18)

This phase of the intervention involves 12 calls over 12 months (one per month) with a focus on maintenance of behaviour change and weight loss. During this phase, participants not yet reaching their initial weight loss, physical activity and/or dietary behaviour change targets are encouraged to continue to work towards these; those who have reached initial targets focus on strategies for maintaining weight loss and behaviour change. In addition to continued support from telephone counsellors, other strategies to promote maintenance include: ongoing self-monitoring, enlisting social support (from family, friends and health care providers), and use of community resources to support lifestyle change (e.g., weight loss groups, walking tracks, gym membership).

### Primary and secondary outcomes

Primary outcomes are: change in weight (kg), accelerometer-measured moderate-to-vigorous physical activity and glycaemic control (HbA1c). Secondary outcomes are: change in self-reported dietary and energy intake, accelerometer-measured light intensity and sedentary time, objectively measured waist circumference, percent body fat, fasting plasma glucose and blood lipids (total cholesterol, HDL-cholesterol, LDL-cholesterol, triglycerides), liver function enzymes, blood pressure, along with self-reported SCE constructs, medication use and health-related quality of life. We will also evaluate the incremental cost-effectiveness of the telephone intervention and examine demographic and health-related moderators of intervention effects and mediators consistent with the underlying theoretical basis. Related measures are described below and summarised in Table 3.

**Table 3:** Summary of Measures

**Anthropometric**	**#**Weight****
	#Waist circumference
	% body fat
	
Cardio-metabolic	**Glycoslyated haemoglobin**
	Fasting blood glucose
	Cholesterol (total, HDL, LDL)
	Triglycerides
	Liver function tests (ALT, AST, LDH, ALP, GGT)
	Blood pressure
	Diabetes medication
	
Behavioural	#**Objectively measured physical activity (PA) via accelerometer**
	Average mod-vig PA/day (mins)
	Average light intensity PA/day (mins)
	Average very light intensity PA/day (mins)
	Average sedentary time/day (mins)
	Breaks in sedentary time (n/day)
	Bouts of moderate-to-vigorous intensity activity (n/day)
	Self-reported physical activity
	Walking (days & min/wk)
	Walking for exercise (days & min/wk)
	Moderate-vigorous PA (days & min/wk)
	Resistance training (days & min/wk)
	Sitting items (transport, television, computer min/wk)
	Self-reported diet
	Dietary intake (total energy, total fat, saturated fat, fibre)
	Dietary behaviour
	Sleep (duration & quality)
	
Psychosocial-Environmental	Depression
	Self-efficacy
	Social support
	Perceptions of the neighbourhood environment
	
Cost-effectiveness	Health-related quality of life
	Health care utilization
	Cost to deliver intervention
	
Intervention Delivery (tracked for each call)	Call duration
	Call completion

All assessed at baseline, 6, 18 & 24 months; #assessed at 12 months; bold text denotes primary outcome

### Data collection

Data are collected via objective measurements conducted in participants' homes, telephone interviews, and self-administered questionnaires at baseline, 6-, 18-, and 24-months by research staff and registered nurses blind to participants' study group. At 12 months, weight, waist circumference and accelerometer-derived physical activity are also assessed. Home visits are conducted by registered nurses and include objective measurement of: height (baseline only; Seca 214 height rod, Seca, Germany), weight, waist circumference, body composition, blood pressure and collection of blood samples for biochemical assay. In addition, details of diabetes medications (drug and dosage) and any use (yes/no) of antihypertensive and lipid-lowering medications are also collected during this visit. Telephone interviews collect self-report data on: demographics (gender, age, education, income, type and number of chronic conditions; baseline only), and validated measures of physical activity, diet, quality of life and health care use, as well as items to assess sleep, diabetes history and management, use of weight loss aids and adverse outcomes. Self-administered questionnaires are used to collect data related to the social-cognitive and environmental constructs which underpin the intervention and to assess depression. In addition to the self-reported physical activity collected via the telephone interviews, accelerometers are used to collect objectively-measured physical activity and sedentary time. Data related to intervention delivery are also tracked by telephone counsellors following each call. This includes data on call outcomes (call completion versus missed calls), call duration and call content (via a checklist of topics). Details of the measures are provided below and summarised in Table 3.

#### Anthropometric outcomes

##### Weight loss

Weight is measured without shoes or heavy clothing to the nearest 0.1 kg using standard calibrated scales (Model TI TBF 350, Tanita Inc., Tokyo, Japan).

##### Waist circumference

Waist circumference is measured with a non-expandable tape to the nearest 0.5 cm at the superior border of the iliac crest [12,72]. Waist measurements are taken in duplicate and a third measurement taken if the first two differ by more than 1 cm. The mean of the measurements is used.

##### Body composition

Fat mass (kg), fat free mass (kg) and percent body fat are measured using foot-to-foot bioelectrical impedance analysis (Model TI TBF 350, Tanita Inc., Tokyo, Japan) in the fasted state. Body composition measurements are not performed on participants with a pacemaker.

#### Cardio-metabolic outcomes

Blood samples are taken early in the morning after an overnight fast (at least 10 hours), with participants instructed not to take any glucose-lowering medication prior to the assessment.

##### Glycaemic control

HbA1c is measured from whole blood samples by the high performance liquid chromatography method (Bio-Rad Variant II, Sydney, Australia). Plasma glucose is measured by the hexokinase method (Roche Modular Analyzer; Tokyo, Japan).

##### Blood lipids

Total cholesterol, high density lipoprotein (HDL) cholesterol and triglycerides are measured by an enzymatic colorimetric assay with Roche Modular Chemistry Analyser (Tokyo, Japan). Low density lipoprotein (LDL) cholesterol is determined using the Friedewald equation [73,74].

##### Liver function tests

Liver enzymes - alanine amino-transferase (ALT), aspartate amino-transferase (AST), lactate dehydrogenase (LDH), alkaline phosphatase (ALP), gamma glutamyl transpeptidase (GGT) - are measured using Roche Modular (Tokyo, Japan).

##### Blood pressure

Blood pressure is measured in the seated position by a portable sphygmomanometer (Gamma G5, Heine, Germany) using the non-dominant arm and an appropriately sized cuff. Two readings are taken and a third measurement taken if the first two differ by more than 10 mmHg systolic or 6 mmHg diastolic, or if the first two readings are more than 140/90 mmHg. Blood pressure is recorded as the mean of the readings taken.

##### Medication use

Details of diabetes medication (oral medication and insulin) are collected by registered nurses from prescription containers - medication name, dose prescribed and daily quantity taken. Participants are also asked to report whether they are taking anti-hypertensive or lipid-lowering medications.

#### Behavioural outcomes

##### Objectively-measured physical activity and sedentary time

A dual-axis accelerometer (Actigraph model GT1M; Actigraph, LLC, Fort Walton Beach, Florida), fitted firmly around the waist by elasticised band and positioned on the right mid axillary line, is used to objectively-derive physical activity and sedentary time. Participants are instructed to wear the accelerometer during all waking hours (removing for any water based activities) for a continuous period of seven days, and to record time worn in a wear-time log. The study nurse demonstrates how to wear the accelerometer and complete the wear-time log. Research staff then telephone the participant to negotiate a start date and reiterate the instructions; the accelerometer and wear-time log are then posted to participants. On the (expected) seventh day of wear, a follow-up telephone call is made by research staff to ensure adequate wear time and to prompt accelerometer return in a reply-paid envelope. The protocol for the follow-up assessment is identical with the exception that the registered nurse does not repeat the demonstration and instructions at the assessment.

Accelerometer data are collected in one minute epochs. A generic program (SAS 9.1), modified from the National Cancer Institute [75], is used to identify outliers and summarise daily variables. The wear time syntax of the program, based on a combination of the wear-time logs and the accelerometer data, is modified for each participant in order to maximise the accuracy of the wear time estimation. Invalid days of observation (days with < 10 hours wear or excessive counts ≥ 20,000 counts per minute: cpm) will be discarded. Daily sedentary (< 100 cpm), very light-intensity activity (100 to 759 cpm), light-intensity activity (760 to 1951 cpm), moderate-intensity activity (1952 to 5724 cpm), and vigorous-intensity activity (≥ 5 725 cpm) [76,77] time are calculated and standardised for wear time using the residuals method [78]. Patterns of physical activity and sedentary time accumulation (for example, breaks in sedentary time [79] and bouts of moderate-to-vigorous intensity activity) [80] are examined. Data are reported as averages for valid days. The data collection and analytic protocol comply with the best-practice guidelines for conducting accelerometer-based activity assessments in field-based research [81].

##### Self-reported physical activity

Self-reported physical activity is measured by the Active Australia Survey (AAS), a 6-item measure assessing frequency and total duration per week spent on walking, vigorous and moderate physical activities over the last seven days [47]. The frequency items have been adapted to ask about number of 'days per week' instead of 'sessions per week'. Total minutes of physical activity is calculated from the sum of walking, moderate and 2 × vigorous minutes and truncated at 1,680 minutes per week [47]. Two items from the U.S. National Health Interview Survey (NHIS) have been adapted to assess the number of days and amount of time in the past week spent walking for exercise [82]. The AAS has been found to have acceptable validity and reliability [83], the NHIS items have shown acceptable validity [82] and both items have shown similar or greater responsiveness to change compared to a more detailed self-report instrument [84].

##### Self-reported resistance training

Using a response format similar to that of the AAS, the number of days and total number of minutes per week of resistance training are collected using an unvalidated item [85].

##### Self-reported sitting time

Six items are used to assess total time spent sitting in the last week across three domains - 1) transport, 2) watching television, videos or playing electronic games and 3) leisure-time computer use - asked separately for weekdays and weekend days. These items have shown good test-retest reliability (unpublished data).

##### Dietary intake

Diet is assessed using the Anti-Cancer Council of Victoria (ACCV) Food Frequency Questionnaire (FFQ) (version 2). The FFQ includes 74 food items and six alcoholic beverage items with responses on a 10-point scale (1 = Never to 10 = 3 or more times per day), four pictorial questions relating to portion size, one question on quantity of alcoholic beverages and 10 cross-check questions used to adjust for overestimation and types of food products consumed (milk, bread, spread and cheese) [86]. It was modified to ask about intake over the previous month instead of the previous 12 months and to be telephone administered instead of self-administered (participants receive a copy of the portion size pictures in the mail). Nutrient intakes are computed from FFQ responses using software developed by the ACCV based on the NUTTAB95 nutrient composition data [87]. The questionnaire estimates intakes of most nutrients accurately (within 10%) and does not systematically under- or over-estimate against weighed records [86].

In addition to the FFQ, dietary behaviours and intakes of specific food groups will also be assessed. Servings of vegetables and fruit are assessed using items from the Australian National Nutrition Survey which have demonstrated validity against more comprehensive self-reported measures [88] and biomarkers (serum carotenoids and red-cell folate) [89]. The 20-item Fat & Fibre Behaviour Questionniare is also administered. This questionnaire was used in our previous trial [40] and adapted from other short dietary questionnaires [90-93] to assess behaviours related to fat and fibre intake that were targeted as part of the intervention. This index has shown good two-week test-retest reliability (ICC = 0.87-0.89), Pearson's correlations of 0.50-0.56 for relative validity compared to FFQ and similar or higher responsiveness compared to FFQ (unpublished data).

##### Sleep

A single item to assess sleep duration on weekdays or workdays in the past week (reported in hours and minutes) was adapted from an existing instrument with established reliability and validity [94-96]. A single item to assess sleep quality (in terms of feeling rested upon awakening; rated from 1 = excellent to 5 = poor) was created.

#### Psychosocial-environmental outcomes

##### Depression

Measured using 9-items from the PRIME-MD Patient Health Questionnaire (PHQ) assessing depressive symptoms over the last two weeks, with items answered on a 4-point scale (0 = not at all; 1 = several days; 2 = more than half the days; 3 = nearly every day). Items are summed such that higher scores indicate a higher depression symptom severity, which has shown good criterion validity compared to a Mental Health Professional interview (*r *= 0.84) [97].

##### Eating and exercise self-efficacy

Measured using a 10-item questionnaire that assesses confidence in ability to follow exercise and eating plans in difficult situations (e.g., confidence in exercising when sore or tired; confidence to choose healthy food options when bored) [98]. Items are answered on a scale from 0 (not at all confident) to 8 (extremely confident), and summed to create a 5-item eating self-efficacy scale and 5-item exercise self-efficacy scale, which have shown good internal consistency - Cronbach's *α *= 0.87 and 0.91, respectively [98].

##### Confidence in weight loss

Using the format and scale above, a single item on confidence in losing weight through exercise and healthy eating was adapted for this study from the Multidimensional Diabetes Questionnaire [99].

##### Social support for diet and physical activity

Measured using a 20-item questionnaire assessing the frequency of family and friend support over the previous three months for healthy eating and exercise (e.g. complimented you on your progress in making healthy changes to your diet; exercised with you); answered on a 5-point scale (1 = none of the time; 5 = very often) [100]; and summed separately for diet and physical activity. The questionnaire was adapted to ask about support received from 'friends, family or members of your household' combined, instead of asking questions separately for support from 'friends' and from 'family.'

##### Perceptions of the neighbourhood environment

Measured using 10-items created to assess neighbourhood attributes related to walking for exercise or transport (i.e., aesthetics, convenience, access and traffic); answered on an 11-point scale (-5 = strongly disagree; +5 = strongly agree, with 0 = neutral option). The items were selected or modified from existing instruments [101,102].

#### Cost-effectiveness

##### Health-related quality of life

The Short-Form (SF)-12 Version 2 Health Survey [103], validated for use in Australia [104,105], is used to assess health-related quality of life across eight dimensions - physical functioning, social functioning, role limitations due to physical problems, role limitations due to emotional problems, bodily pain, vitality, general health and mental health. To enable economic analysis, the SF-12 is converted to EQ-5 D preference based utility values via an algorithm developed by Gray and colleagues [106].

##### Health care utilisation

Measured using 9-items assessing doctor and allied health care visits and hospital stays in the past six months [107]. Dollar valuations of these resources are obtained from the Commonwealth Government schedule of re-imbursements [108] and are used in cost-effectiveness analysis.

##### Intervention costs

Costs to deliver the intervention, not including the research/assessment components, are used in the cost-effectiveness analysis. They are tracked during trial implementation and include the cost of counsellor time, intervention materials (workbook, pedometer, stretch band, scale) and related infrastructure (i.e., office space, telephones, computers and call costs).

### Patient safety

A number of strategies are used to ensure patients' safety. Intervention participants who identify new health concerns or questions about the medical management of diabetes are recommended to visit their GP to seek advice. Any participant who indicates suicidal ideation on the PHQ-9 scale is contacted by the project manager and informed that their GP will be notified and a 24-hour telephone crisis service number is provided. Adverse outcomes are also systematically monitored, as described below.

#### Adverse outcomes monitoring system

Adverse outcomes are assessed at each follow-up telephone interview by asking participants if they have had any new health problems since the previous contact, with the following examples provided: any new diagnoses of medical conditions or illnesses, such as a heart attack or cancer, any periods of hospitalisation or surgery, any muscle injuries, bone or joint problems, or any new symptoms or worsening of pre-existing conditions. Responses are recorded verbatim and then coded independently by two of the study investigators, first as "serious" (i.e., requiring hospitalisation) versus not, and then as "directly attributable to the study" (i.e., exercise-related pain or muscle injury, digestive disturbances related to dietary changes) versus not, with input on disagreements discussed with a researcher independent of the trial.

### Statistical analyses

#### Primary and secondary outcomes

Data will be analysed using intention-to-treat principles. Once a participant is randomised into a study group, they will be considered a trial participant and analysed according to their allocated group, regardless of missing data for follow-ups or the amount of intervention received. Each primary and secondary outcome will be modelled using mixed linear models with random intercepts, the fixed effects of study group, time and a group by time interaction as well as baseline values (to account for regression to the mean). Potential confounders include baseline characteristics and the factors used to balance groups by minimisation. Confounders will be identified by forwards and backwards selection. Inclusion in the final model will require the addition or omission of the potential confounder to elicit a substantial change in the coefficient for the effect of intervention. A 20% change in coefficient will be considered substantial unless the coefficient is close to zero. Data analysis will be performed using [SAS/STAT] software, Version [9.2] of the SAS system for Windows. Statistical significance will be set at the conventional two-tailed 5% level.

#### Incremental cost-effectiveness

A probabilistic decision analytic Markov model developed for our recent telephone-delivered lifestyle intervention trial will be adapted and used to evaluate incremental cost-effectiveness [42]. The structure, data requirements, methods for calibration and model evaluation are described in a recent methodological peer reviewed publication [91]. The model describes the health states relevant to intervening on weight loss, physical activity, and glycaemic control in adults with type 2 diabetes. A societal perspective is adopted and values collected for variables that describe the participant's use of acute and primary care health services, their private out of pocket expenditures and the time costs to informal carers. Patient-based production losses are not included on the cost side of the analysis but (implicitly) counted within the QALY estimation in line with current theory and recommendations [92]. The model implies the trial participants face some probability of remaining in a sub-optimal health state or transitioning to improved health states (i.e. improvements in weight, physical activity and glycaemic control) and there will be a small chance of transitioning to death. Cycle duration is based on the timing of data collection for health-related quality of life (i.e. baseline, 6, 18, and 24 months). The transition probabilities that reflect consistency with study intervention targets surrounding primary outcomes (i.e., 5% weight loss, 210 min/wk moderate-to-vigorous PA, < 7% HbA1c) will be used to predict movement through the states for all cycles; this approach is shown in a previous economic analysis [42]. Costs and utility based preference scores will be estimated for each cycle and summarised to estimate change in costs and change in QALYs. The relevant ICER will be estimated and cost-effectiveness acceptability curves plotted to illustrate the probability that the intervention is cost-effective [109].

#### Moderators and mediators of outcome

Exploratory analyses will be conducted to determine whether there is moderation or mediation of intervention effects using previously established methods [95,96]. Moderator analysis will determine whether intervention effects differ across demographic (e.g. age, gender) and medical (e.g. insulin use) characteristics and will be performed by considering the statistical significance of an interaction between a potential moderator and the intervention using a Wald test.

Mediator analysis will determine whether theoretically-driven constructs and mechanisms for behaviour change do in fact mediate the intervention effects. Potential mediators will be assessed using path analysis. Point estimates and bootstrap confidence intervals of path coefficients and the product of the mediated path coefficients will be used to determine the potency, certainty and direction of any mediation effect [110].

### Sample size

Effect size estimates for the proposed study take into account clinically meaningful differences and are based on published results [24,111,112], and unpublished data from our recent trials. The minimum differences of interest in change from baseline to 18-months follow-up between intervention and usual care groups for our primary outcomes are: -5% of initial body weight; +60 minutes/week for moderate physical activity; -0.6% for HbA1c. Sample size estimates are based on the expected mean difference for physical activity, as this variable required the largest sample size. To detect a mean difference of 60 minutes per week change in accelerometer-derived moderate-to-vigorous intensity physical activity between the intervention and control groups with 90% power and 5% significance (two-tailed), assuming a standard deviation of 140 minutes (based on unpublished data), requires 115 subjects per group. Assuming an attrition rate of 30% means that 150 participants per group are required (300 participants overall). This sample size provides sufficient power (80% or more) to detect the following intervention effects for sedentary time (-45 minutes/day); light intensity time (+30 minutes/day); and, our secondary outcomes: -2,000 kJ for energy intake; -5% for energy from fat; -2% for energy from saturated fat; -5 to 10% for cholesterol (total, LDL-, HDL-); -15% for triglycerides; -1 mmol/L for fasting plasma glucose; -5 mmHg for systolic blood pressure; -5 cm for waist circumference; and, -2% for body fat.

## Discussion

The epidemic of type 2 diabetes shows no signs of abating. Both weight loss and physical activity are independently associated with improved glycaemic control, lipids and blood pressure in type 2 diabetes [49] and are important diabetes management goals. Thus there is a need to develop, evaluate and disseminate approaches that are effective in promoting the maintenance of weight loss and physical activity. Telephone-delivered interventions show promise in this regard as they facilitate, in a cost-effective manner, [42] the repeated contacts and support necessary to promote maintenance.

The Living Well with Diabetes study is evaluating a broad reach strategy (telephone counselling), with emphasis on behavioural and physiologic outcomes, fidelity of implementation, incremental cost-effectiveness, and assessment of maintenance. These issues are important to advancing the science of mass reach approaches to weight management and health behaviour change, as well as building the evidence base for the translation of this work into population health practice.

## Competing interests

The authors declare that they have no competing interests.

## Authors' contributions

All authors contributed to the design of the study and the writing of the manuscript. All authors read and approved the final manuscript.

## Pre-publication history

The pre-publication history for this paper can be accessed here:

http://www.biomedcentral.com/1471-2458/10/452/prepub
